# Histopathological analysis of T1 renal cell carcinoma: Does presentation matter?

**DOI:** 10.4103/0970-1591.44257

**Published:** 2008

**Authors:** Gaurav Gupta, Samiran Das Adhikary, Santosh Kumar, Ninan K. Chacko, Nitin S. Kekre, Ganesh Gopalakrishnan

**Affiliations:** Department of Urology, Christian Medical College and Hospital, Vellore, Tamil Nadu - 632 004, India

**Keywords:** Incidental, nephron-sparing surgery, renal cell carcinoma

## Abstract

**Objectives:**

To study the differences in the clinico-pathological features of incidental and symptomatic T1 renal cell carcinoma (RCC) and to see, particularly in T1b RCC, if symptomatic presentation has adverse pathological features concerning the oncological safety of elective nephron-sparing surgery (NSS) in this subgroup.

**Materials and Methods:**

Of 278 patients who underwent radical nephrectomy for RCC from January 1995 to January 2005, 70 had tumor size up to 7 cm (T1). They were categorized as incidental or symptomatic and as T1a or T1b tumors. Clinico-pathological features were compared between incidental (IRCC) and symptomatic (SRCC) groups. Tumors were analyzed using the 1997 TNM staging and Fuhrman's grade.

**Results:**

Of the 70 with T1 tumors, 24 had T1a (IRCC, 12 and SRCC, 12) and 46 had T1b tumors (IRCC, 27 and SRCC, 19). Clear cell was the commonest histology. In T1a cancers, though no significant difference in histopathological pattern and grade was seen between the incidental and symptomatic groups, symptomatic tumors had more papillary, mixed histopathological pattern and higher nuclear grade. Among T1b tumors, 14 had papillary and mixed histology, 12 (86%) of which were symptomatic (*P*= <0.0001). In T1b, 15 (79%) symptomatic had higher nuclear grade (G2-3) while 22 (81%) incidental had lower Fuhrman′s grade (*P*= <0.0001).

**Conclusion:**

Symptomatic T1b RCCs had higher nuclear grade and papillary histology. This difference was statistically significant. This may be relevant when considering elective NSS in symptomatic T1b disease.

## INTRODUCTION

Renal cell carcinoma (RCC) accounts for approximately 2% of adult malignancies and 80-85% of malignant kidney tumors.[1] With the increasing use of noninvasive imaging in those with nonspecific symptoms, more than 50% of the RCCs are detected incidentally in younger age and are now referred to as the “radiologist's tumor”.[1,2]

Studies have demonstrated that incidental RCCs tend to be smaller, lower-stage and with better survival outcomes as compared to symptomatic RCC.[3] Although the contributions of lead and length time bias have not been defined, others have challenged this claim.[4,5]

With the evolution of nephron-sparing surgery (NSS), open or laparoscopic partial nephrectomy has become the standard of care. While absolute indications for NSS are well accepted, the most controversial in current practice are the elective indications. Based on existing data, elective partial nephrectomy is usually indicated for a renal mass less than 4 cm in size, peripherally located and easily resectable.[6–8] Recently, several investigators have questioned the conventional 4 cm size cutoff for elective partial nephrectomy[9] and have extended its application to T_1b_ tumors.

While studying T1 RCC we were surprised at some features of its clinico-pathological profile. This has made us raise concerns about the oncological safety of NSS in T_1b_ cancers.

## MATERIALS AND METHODS

This retrospective review was conducted from January 1995 to January 2005. Of 278 patients who underwent radical nephrectomy, 70 (25%) with T1 stage formed the study group. Those with familial and bilateral RCCs were excluded from the study. Patients were categorized according to their clinical presentation. Tumors diagnosed incidentally as a part of a health checkup or for evaluation of vague upper gastrointestinal symptoms comprised the incidental (IRCC) group. Patients presenting with loin pain, hematuria, weight loss, cachexia and mass formed the symptomatic (SRCC) group. Of the 70 patients, 39 (56%) and 31 (44%) were in IRCC and SRCC groups respectively.

The histopathological data was retrieved from the pathology database. Staging was based on TNM, 1997 classification. For grading Fuhrman nuclear grade was used. Both groups were compared for clinical and pathological parameters with mean follow-up of 36 (12-108) and 60 (12-120) months in IRCC and SRCC respectively.

### Statistical analysis

All the categorical variables were summarized using frequencies and percentages. Significance was calculated using Fisher's exact test. A two tailed *P*-value < 0.05 was considered as statistically significant.

## RESULTS

### Patient characteristics

The mean age in the IRCC and SRCC group was 52.6 (32-76 years) and 52.4 (21-76 years) years respectively. Female preponderance was seen in incidentally detected RCC (46%), as compared to the symptomatic group (16%). In the symptomatic group the majority presented with hematuria [Table 1].

**Table 1:** Presentation in SRCC group (n=31)

Symptoms	No (%)
Hematuria	28 (90)
Mass	01 (03)
Pain	12 (39)
Weight loss	08 (26)

### Tumor characteristics

Patient distribution in IRCC and SRCC is shown in Table 2. Pathological examination of operative specimen of T_1a_ group did not reveal a statistical difference for different histological patterns (clear cell vs. papillary and mixed; *P*=0.37) and grade (Grade-1 vs. Grade 2-3; *P*=0.09). In the T_1b_ group, compared to the clear cell type, papillary and mixed histological varieties were significantly more in the SRCC than IRCC group (*P* <0.0001). Symptomatic T_1b_ RCCs had a significantly higher grade (2-3) [Table 3] than Grade-1 (*P* <0.0001).

**Table 2:** T1 stage and sub-classification of IRCC and SRCC

Stage	IRCC, no (%)	SRCC, no (%)
T1	39 (56)	31 (44)
T1a (≤ 4cm)	12 (50)	12 (50)
T1b (> 4-7cm)	27 (59)	19 (41)

**Table 3:** Histopathological characteristics in Incidental and Symptomatic renal cell carcinoma

Histological pattern and Grade	T1a (n=24)			T1b (n=46)		
		
	IRCC (n=12)	SRCC (n=12)	*P*	IRCC	SRCC (n=27)	*P* (n=19)
Clear cell (%)	10 (59%)	07 (41%)		25 (78%)	07 (22%)	
Papillary (%)	01 (25%)	03 (75%)	0.37	02 (17%)	10 (83%)	0.0001
Mixed (%)	01 (33%)	02 (66%)		00 (0%)	02 (100%)	
Grade-1 (%)	09 (75%)	04 (25%)		22 (85%)	04 (15%)	
Grade-2 (%)	03 (30%)	06 (70%)	0.09	04 (29%)	10 (71%)	0.0001
Grade-3 (%)	00 (0%)	02 (100%)		01 (17%)	05 (83%)	
Mean tumor	3.5 (3.5-4)	3.7(3-4)	n.s.	5.6 (4.5-7)	5.5 (5-7)	n.s.

Size in cm (range), n.s.: not significant

One of the patients in SRCC T_1b_ had sarcomatoid variant of clear cell type. One in T_1a_ SRCC (8%) and two in SRCC T_1b_ (11%) group developed distant metastasis at 64, 34 and 48 months of follow-up respectively but none developed in the IRCC group. In SRCC T_1a_ and T_1b_ the five-year cancer-specific survival rate was 100 and 89%. While in IRCC T_1a_ and T_1b_ it was 100%. The mean follow-up in IRCC and SRCC was 36 (12-108) and 60 months (12-120 months) respectively.

## DISCUSSION

Renal cell carcinoma is characterized by diverse clinical manifestations. Small, localized tumors rarely produce symptoms and for this reason the diagnosis is often delayed. Symptomatic presentation is usually a sign of advanced disease. The most common presentations are hematuria (50-60%), abdominal pain (40%), and a palpable mass in the flank or abdomen (30-40%). These three symptoms occur as a combination (“classic triad”) in less than 10% of patients.[10] Other signs and symptoms are relatively nonspecific — fever, night sweats, malaise, and weight loss. In our series hematuria was the most common presentation, which is intuitively a sign of pelvi-calycial system invasion and makes metastasis disease more possible. Several studies have demonstrated that incidental neoplasms tend to be smaller, lower stage and grade and have better survival outcomes when compared with symptomatic RCC.[3] On analyzing our results we also found that incidental tumors were low-grade and with a favorable histology as compared to the symptomatic tumors. Licht *et al.*, found that symptomatic renal tumors (>4 cm) treated with partial nephrectomy had a statistically significant worse prognosis. In their series five-year cancer-specific survival rates for incidental and symptomatic RCC was 94 and 83%.[11] This prompted Patard *et al.*, to propose a classification based on mode of presentation (incidental or symptomatic) combined with tumor size to stratify prognosis.[12]

Renshaw *et al.*, found that papillary RCCs were more aggressive as compared to clear cell RCC.[13] In T_1a_, seven patients had papillary and mixed histology, of which five were symptomatic. In T_1b_, papillary and mixed histology was seen in 14 patients, of which 12 were symptomatic (*P*=0.0001; Table 3). Kletscher *et al.*, have shown that multi-focality occur at a significantly higher rate (*P*=0.011) with papillary and mixed histological pattern.[14] Sarcomatoid histology is no longer considered a distinct subtype of RCC. It represents a relatively rare, poorly differentiated form of RCC subtypes; seen in less than 5% of RCCs and associated with dismal prognosis.[15] In our study sarcomatoid differentiation was seen in one patient who had a symptomatic presentation.

Ghavamian *et al.*, in a multivariate model reported, tumor stage and nuclear grade were significantly associated with death from RCC.[16] Castilla *et al.*, reported significant risk of disease progression with increasing Fuhrman nuclear grade.[17] In Fuhrman's original report, the five-year survival rates for Grades 1 to 4 were 64%, 34%, 31% and 10% respectively. Nuclear grade proved to be the most significant prognostic factor for Stage-I tumor in his series.[18] In our study, 15 in the SRCC group as compared to five in the IRCC of the T_1b_ had higher nuclear grade (G2,3) [Table 3]. Fergany *et al.*, have shown a significant survival benefit not only for smaller lesions but also for those who had incidental presentation and with lower grade.[6]

Elective NSS has become standard treatment for T_1a_ tumors. The rate of local recurrence in partial nephrectomy varies from 0-10%[19] and reflects the fact that most patients treated in this manner had large tumors, multi-focal tumors (microscopic/macroscopic), or both. Reported overall survival of NSS is similar to that of patients with disease of similar stage (T1) when compared with radical nephrectomy.[6,8] In the era of advanced laparoscopic surgery technical success is excellent and operative morbidity and mortalities are low.[19] In contemporary series cancer-specific survival has been found to be between 95-100 %.[19] Because of these favorable results in T_1a_ RCCs and increased number of diagnosis at early stage of tumors, elective NSS approach has been extended to select exophytic >4-7 cm size RCCs be they symptomatic or incidental. This is in spite of large partial nephrectomy series, showing size to be the most significant predictor of cancer-related outcome [Table 4].[6,7,9,11]

**Table 4:** Disease-free survival in patients after NSS evaluated by tumor size

Study	Total No. of patients	Number <4 / 4-7 cm	Five-year cancer-specific survival (%)		Electively done (%)
				
			<4cm	4-7cm	
Fergany *et al.*[6]	107	43 / 21	98	95	02
Hafez *et al.*[7]	485	240 / 80	96	86	09
Belldegrun *et al.*[9]	108	53 / 10	100	90	58
Lich *et al.*[11]	216	124 / 82	97	79	08

Rational for expanding elective NSS to larger tumors include recent changes in the staging of RCC suggesting a favorable tumor-related prognosis for individuals with tumors less than 7 cm, increase in the life expectancy, incidental diagnosis at an earlier age and metachronous tumor recurrences in patients undergoing radical nephrectomy and thus increased concerns about the long-term risk of renal insufficiency. Our analysis on tumors > 4 cm in size has shown that symptomatic RCCs were associated with higher nuclear grade and papillary histology; the latter is associated with multi-centricity and could account for lower disease-free survival in T_1b_ RCCs as reported in the literature. This data indicates that it may be a cause for concern for elective NSS in those who had symptomatic presentation.

In this context we feel that there could be a role of core needle biopsy in the management decision for symptomatic T_1b_ RCC. In a review by Lane *et al.*, renal mass biopsy has a 94% overall accuracy for identifying RCC subtypes.[20] Fuhrman nuclear grade has been assessed correctly up to 83% on these biopsies. These discrepancies in grades are likely due to tumor heterogeneity, since 16-25% of renal tumors show some variability of grade.[21] Hence to enhance the yield, it is important to obtain samples from various areas of the mass.[20] In conjunction with conventional histological analysis of renal mass biopsies, using core needle biopsy (18-gauge needle) with minimal or no increase in risk of complications,[20] additional tests such as immuno-histochemistry, molecular markers, fluroscent *in-situ* hybridization[22] and polymerase chain reaction[23] further improve the accuracy of these biopsies.

This is a retrospective analysis with small numbers. Further prospective studies are required to analyze the adverse factors of elective NSS in symptomatic T_1b_ RCCs.

## CONCLUSION

Symptomatic RCCs had a higher nuclear grade and unfavorable histology. In partial nephrectomy the main worry is of local tumor recurrence and metastasis thus affecting the disease-free survival. We have found that symptomatic T_1b_ RCC had an unfavorable histology and higher grade both of which are known to be associated with multi-centricity, higher recurrence rate and poor prognosis. These data indicate that NSS should be avoided in symptomatic RCCs but could be extended to select incidentally diagnosed 4-7cm-sized renal cell carcinomas.
